# A repeatable scoring system for assessing Smartphone applications ability to identify herbaceous plants

**DOI:** 10.1371/journal.pone.0283386

**Published:** 2023-04-05

**Authors:** Neil Campbell, Julie Peacock, Karen L. Bacon

**Affiliations:** 1 Botany & Plant Science, School of Natural Science, University of Galway, Galway, Ireland; 2 School of Geography, University of Leeds, Leeds, United Kingdom; University of Palermo, ITALY

## Abstract

The ubiquity of Smartphone applications that aim to identify organisms, including plants, make them potentially useful for increasing people’s engagement with the natural world. However, how well such applications actually identify plants has not been compressively investigated nor has an easily repeatable scoring system to compare across plant groups been developed. This study investigated the ability of six common Smartphone applications (Google Lens, iNaturalist, Leaf Snap, Plant Net, Plant Snap, Seek) to identify herbaceous plants and developed a repeatable scoring system to assess their success. Thirty-eight species of plant were photographed in their natural habitats using a standard Smartphone (Samsung Galaxy A50) and assessed in each app without image enhancement. All apps showed considerable variation across plant species and were better able to identify flowers than leaves. Plant Net and Leaf Snap outperformed the other apps. Even the higher preforming apps did not have an accuracy above ~88% and lower scoring apps were considerably below this. Smartphone apps present a clear opportunity to encourage people to engage more with plants. Their accuracy can be good, but should not be considered excellent or assumed to be correct, particularly if the species in question may be toxic or otherwise problematic.

## Introduction

Plants account for over 80% of biomass on the planet [1] but many people struggle to identify even common plants in their own areas [2–5]. This lack of engagement with plants is often referred to as “plant blindness”, although “plant awareness disparity” [6] and “plant awareness” [5, 7] have both been suggested as more inclusive terms to refer to the same phenomenon. Problems associated with a lack of ability to “see” and identify plants has also manifested in conservation [8–10] with rosewood being noted as one of the most trafficked organisms (Margulies et al. 2019) [11] and large numbers of cacti being trafficked in 2021 [12]. The ongoing decline in taxonomic and botanical training in many universities [7] further increases the decline of people with knowledge of plant identification, which presents problems for a wide-range of areas—directly for areas such as environmental consultancy and indirectly for areas such as city planning, medicine and teaching [7].

Technology offers a considerable opportunity to engage people with plants [13] and one potential means of increasing the general engagement of people with plants that is straight-forward is the use of Smartphone Applications (hereafter apps). The number of people with smartphones has increased exponentially over the last few years with approximately 14.91 billion smartphones estimated to be in circulation in 2021 [14]. Almost all Smartphones have at least one app that can aid in identifying a wide range of things, including plants, pre-installed—Google Lens. A wide variety of other identification apps also exist, some generalist and some specialised on plants. Some effort has been made to determine how effective various apps are at identifying plants [e.g. 15–17]; but results are variable and sometimes difficult to reproduce. The aim of this study was to comprehensively investigate six popular apps to determine how well they can be used to identify common herbaceous angiosperms using the Irish flora as a test case and to provide a clear and easily reproducible scoring system to compare between apps.

## Materials and methods

### App selection

Six popular, free to download and use ID apps that can be installed on mobile smartphones were selected for evaluation in this study. Table 1 lists the apps and provides details on their popularity at the time of download (spring 2021). Plant Net, Leaf Snap and Plant Snap were among the top four suggestions in the Google Store, Google Lens was selected due to its pre-installation on virtually all devices and its link to the widely used Google Search engine [18]; and Seek and iNaturalist, which both utilise the same database, were created through collaboration of the National Geographic Society and the California Academy of Sciences [19]. iNaturalist relies on a database and requires users to confirm identifications but can also provide “live” identification and has been used in this manner for this study. Certain apps advertise premium versions that offer additional features such as the removal of ads (Leaf Snap). Plant Snap limits the user to five free identifications per day; therefore, a premium version of the app was purchased in order to carry out the study. Generalist apps such as Google Lens, can perform a variety of functions including identifying plants, animals, insects, text, items and products. Specialist apps are those focused on identifying plants only, such as Plant Net and Leaf Snap. All apps provide some form of information about the plants in identifications suggested to the user. Information provided by the apps varies between apps but can include plant taxonomy, morphology, native range, habitat, advice on how to grow certain plants, where to purchase seeds and more. Some of this information is stored in-app and some is sourced directly from internet sources such as Wikipedia. Plant Net and Leaf Snap require the user to specify the plant organ (e.g. leaf or flower etc) and Plant Net, Plant Snap and iNaturalist require the user to confirm the identification.

**Table 1:** Plant identification applications selected for evaluation for this study with ratings and approximate number of downloads as of November 2021.

App	Link	Rating on Google Play Store	Rating on Apple App Store	App “type”
Google Lens	Lens.google	4.5/5 stars (1M Reviews) 1B+ Downloads	N/A	Generalist
Plant Net	https://identify.plantnet.org/	4.6/5 stars (187K Reviews) 10M+ Downloads	4.7/5 stars (1K Reviews)	Specialist
Plant Snap	Plantsnap.com	3.4/5 stars (93K Reviews) 10M+ Downloads	4.6/5 stars (12k Reviews)	Specialist
Leaf Snap	https://leafsnap.app/	4.9/5 stars (24K Reviews) 1M+ Downloads	4.8/5 stars (989 Reviews)	Specialist
Seek	https://www.inaturalist.org/pages/seek_app	4.3/5 stars (5K Reviews) 1M+ Downloads	4.8/5 stars (2.5k Reviews)	Generalist
iNaturalist	https://www.inaturalist.org/	4.2/5 stars (6K Reviews) 1M+ Downloads	4.7/5 stars (225 Reviews)	Generalist

App ratings and download numbers were obtained from the Google play store and the Apple app store. The Apple app store does not display any information on the number of downloads each app has received. Apps are listed as either generalist or specialist depending on whether they were developed to identify plants (specialist) or are a more general identification app.

### Data collection

The Irish flora is relatively depauperate compared to other European countries due to geographic, climatic and ecological reasons [20]. The flora consists of many cosmopolitan species with wide distributions in Europe and Asia but associations of species that are relatively uncommon elsewhere. For example, plants that otherwise have an artic or alpine distribution can be found growing together or in close proximity to each other in the Burren in the West of Ireland. This makes the general flora a useful test case for plant ID apps. Photographs were taken at four sites: the Burren Nature Sanctuary, Kinvarra; Connemara National Park, Letterfrack and University of Galway campus all in Co. Galway and the National Botanic Gardens in Dublin. In total, 38 species across 15 families were included in the study (S1 Data).

All images were captured in the field on a Samsung Galaxy A50 camera with no image enhancing aids such as external lenses or tripods. Images were taken against natural backgrounds to replicate real-world use of the apps to provide an accurate assessment of their capabilities. Where flowers were very small (e.g. Common Mouse-ear Chickweed (*Cerastium fontanum*)) or when it was windy, stems were held in hand to allow for a picture to be taken. Photographs were taken of leaves and flowers from either five or ten different individuals for each species and each photograph was then run through each app.

### Scoring systems

Two scoring systems were developed based on the functionality of the selected apps. Scoring system A focused on how well the app could identify the species based on the first five options provided, while the other, scoring system B allowed points to be scored only if the first answer was correct to species level.

For scoring system A (Table 2), points were awarded based on successful identification of a species, genus, or family. Additional points were awarded based on the placement of the correct identification in the list of options provided by the app. To generate a score for the photograph, points for identification and points for the order in which the correct identification was shown were added together. For example, if a photograph provided a correct species-level identification it was awarded 3 points and if that was the third option provided it was awarded another 3 points, giving a total of 6 points to the app. Had it provided a correct identification on the first option, then it would score 8 points.

**Table 2:** Scoring system A. Scores were allocated to a plant organ image (leaf or flower) by combining the taxonomic score and the image placement score.

Taxonomic score		Image placement score	
Species	+3	Image 1 correct	+5
Genus	+2	Image 2 correct	+4
Family	+1	Image 3 correct	+3
**Incorrect/no identification to family level**	0	Image 4 correct	+2
		Image 5 correct	+1
		**Later Images Correct**	0
Final Score = Taxonomic Score + Image Placement Score			

Scoring system B was developed because some apps, notably Seek in this study, only provide one suggestion. The “1/0” scoring system helped to reduce bias against apps that produce a single suggestion, and highlighted which apps produced correct species identification on the first attempt. A point was awarded only if the app provided the correct, species-level identification on the first attempt.

### Statistical analysis

Statistical analysis was conducted in Past version 4.07 (https://www.nhm.uio.no/english/research/infrastructure/past/). The scoring system led to categorical data that strongly deviated from normality.

Scores for each of the images captured for this study were analysed as a whole with N = 10 flower images and N = 10 for leaf images for the families Asteraceae, Fabaceae and Rosaceae, N = 5 flowers and N = 5 leaf images for the remainder of the species (S1 Data). The scores for each organ per species were averaged to give a single score for flowers and leaves of each species per app. Shapiro-Wilk Normality tests were conducted to determine if the data were distributed normally. Subsequently, Bonferroni-corrected Mann-Whitney U pairwise tests were conducted. Kruskal Wallis tests were carried out for each set of data (leaves and flowers for each species, for each family and across all 38 species in total). Corrected H values and p values for each Kruskal Wallis test were also recorded. All raw data, descriptive data, and the scores generated from the analysis and scoring system are presented in boxplots in the S1 Data.

Chi-square tests were conducted on each data set from scoring system B (Correctly identified leaves and flowers vs. incorrectly identified). The results were analysed and a percentage value of correctly identified images to a species level were recorded and plotted in pie charts. Raw data is presented in S1 Data.

## Results

### Scoring system A

**Fig 1:**
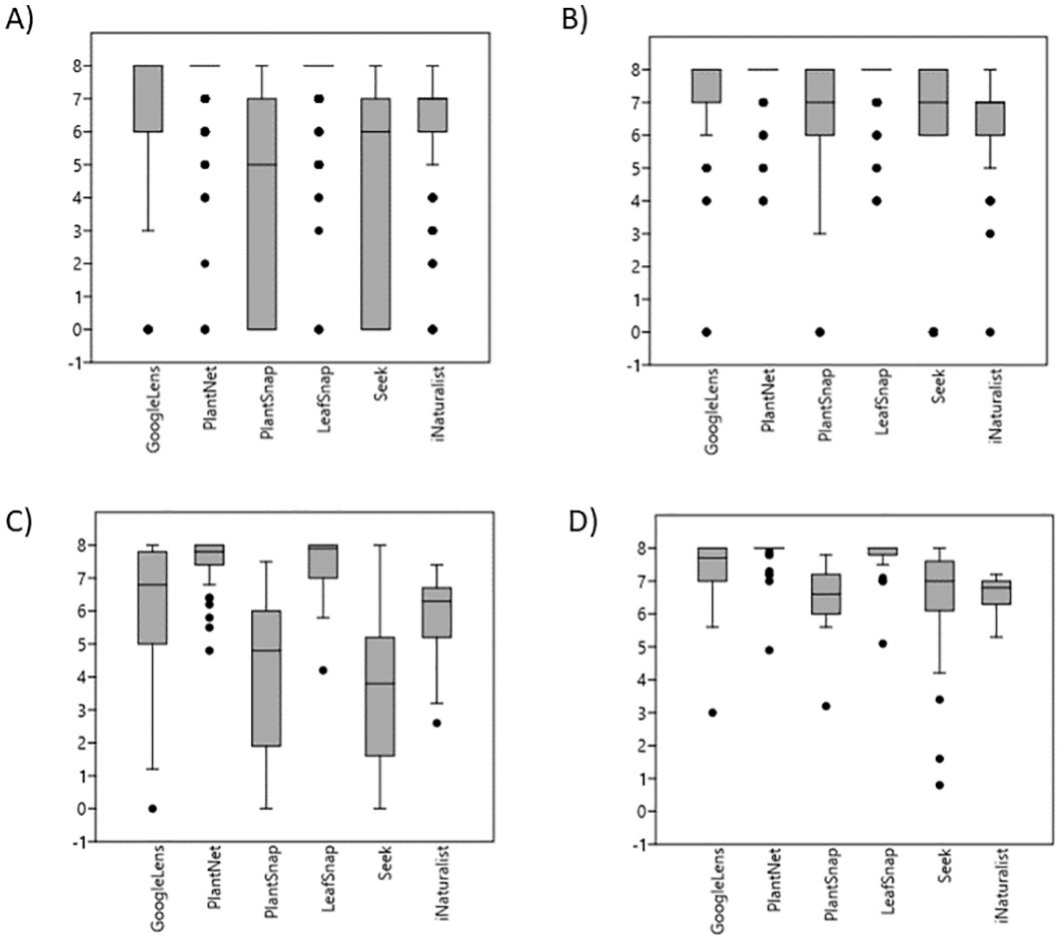
Leaf and flower scores across the entire data set for each plant ID app using scoring system A. (A) Leaf raw data scores (e.g. each individual score per photograph is included) N = 280 (B) Flower raw data scores N = 280 (C) Leaf Average Scores (one score averaged across all photographs per species) N = 38 (D) Flower Average Scores N = 38.

Considerable variation in accurate species identification was observed between apps and between leaves and flowers of the species assessed. Apps were better able to identify species based on images of flowers than leaves and Plant Net and Leaf Snap (Fig 1) preformed best across both leaves and flowers.

Although Plant Net and Leaf Snap are the highest scoring apps across the entire dataset, there was considerable variation in the individual species scores (S1 Data) which is clearly visible with the extent of the whiskers in the box plots (Fig 1). Seek and Plant Snap score considerably lower than the other apps.

Plant Net and Leaf Snap scored significantly higher than the other apps (p < 0.003; Fig 1) but did not score significantly better or worse than each other for leaves or flowers in both raw and average datasets (p = 1). In the raw data, Plant Snap scored similarly to Seek for leaf images (p = 1) and similarly to iNaturalist for flower images (p = 0.9066). Aside from these cases, Google Lens, Plant Snap, Seek and iNaturalist scored significantly differently to each other (p < 0.001).

When the data for each species organ was averaged, it was found that Google Lens and iNaturalist identified leaves with similar levels of success (p = 0.6208), as did Plant Snap and Seek (p = 1). Averaged data for flower identification showed that Google Lens scored similarly to Seek (p = 0.1733) but scored significantly higher than Plant Snap and iNaturalist (p < 0.0001). Seek did not score significantly worse than Google Lens (p = 0.1733) or significantly better than Plant Snap and iNaturalist (p > 0.75). Plant Snap and iNaturalist did not show a significant difference in flower identification scores when the score was averaged per species (p = 1).

Table 3 shows key descriptive statistics for the leaf and flower analysis of both raw and averaged data for each app. Plant Net and Leaf Snap recorded the lowest variance, the highest mean score and the highest minimum score for leaf and flower raw data and averaged data. Seek and Plant Snap recorded the lowest mean score and the highest variance for leaf and flowers in the raw data, and the averaged data. All apps achieved a maximum score of 8 for some species and so this is not shown in the table. Plant Net and Leaf Snap scored the highest minimum score in the raw flower dataset. The minimum scored achieved by all apps in the raw leaf dataset was 0 points. This contrasts to the highest score achieved in the raw flower dataset which was 4 points for both Plant Net and Leaf Snap.

**Table 3:** Key descriptive statistics recorded for both raw and averaged data for both the leaf and flower datasets.

			Google Lens	Plant Net	Plant Snap	Leaf Snap	Seek	iNaturalist
Leaves	Raw data	Variance	7.20	1.73	9.02	**1.68**	12.92	3.96
		Mean	6.40	**7.54**	4.49	7.50	3.91	5.99
		Minimum	0	0	0	0	0	0
	Averaged data	Variance	4.52	**0.64**	5.15	0.76	5.70	1.41
		Mean	6.12	**7.50**	4.15	7.46	3.65	5.92
		Minimum	0	**4.8**	0	4.2	0	2.6
Flowers	Raw data	Variance	2.36	0.62	3.29	**0.54**	7.11	1.54
		Mean	7.27	**7.75**	6.41	7.73	6.36	6.45
		Minimum	0	**4**	0	**4**	0	0
	Averaged data	Variance	1.08	0.32	1.20	**0.29**	2.55	0.42
		Mean	7.26	**7.80**	6.45	7.77	6.51	6.54
		Minimum	3	4.9	2.5	**5.1**	1.6	4.3

The lowest variance, highest mean score and highest minimum scores are shown in bold.

### Scoring system B

Scoring system B yielded similar results to scoring system A with Plant Net again scoring best. Percentages of accurate species identifications are shown in Table 4.

**Table 4:** Percentages of leaves and flowers where the first suggestion is a correct species identification (N = 280).

Flowers	Google Lens	Plant Net	Plant Snap	Leaf Snap	Seek	iNaturalist
% Correct	67.86	**88.21**	35.71	83.57	52.14	3.57
% Wrong	32.14	**11.79**	64.29	16.43	47.86	96.43
Rank	3	**1**	5	2	4	6
Leaves	Google Lens	Plant Net	Plant Snap	Leaf Snap	Seek	iNaturalist
% Correct	55.00	**80.36**	17.14	77.14	22.86	6.79
% Wrong	45.00	**19.64**	82.86	22.86	77.14	93.21
Rank	3	**1**	5	2	4	6

The highest performing app (Plant Net) is shown in bold.

Plant Net and Leaf Snap were the highest scoring apps of those selected for this study. Plant Net scored significantly more species correctly than incorrectly (*p* = 4.9716^e-33^), identifying 88.21% of flowers and 80.36% of leaves (*p* = 6.644^e-42^) to a species level on the first suggested identification. Leaf Snap was the next best performing app, correctly identifying 83.57% of flowers (*p* = 1.50^e-24^) and 77.14% of leaves (*p* = 4.5023^e-35^) to species level. iNaturalist performed the worst with scoring system B. iNaturalist provided a significant number of incorrect identifications of flowers and leaves (*p* = 1.3509^e-79^ and *p* = 2.473^e-40^ respectfully) with 3.57% of flowers and 6.79% of leaves successfully identified to a species level at the initial suggestion. This is due to iNaturalist often preferentially providing a genus identification rather than a species identification. Seek, which only returns a single suggestion for each image and preformed worst in scoring system A, achieved the lowest mean score (with the exception of the mean score of the averaged flower data), the lowest minimum score and the highest variance for both flowers and leaves. Seek correctly identified 52.14% of flowers, which was not significant (*p* = 1), and a significant number of leaves were incorrectly identified (*p* = 5.96^e-13^), with only 22.86% correctly identified to a species level.

### Flowers v leaves

Overall, flowers are more easily identified than leaves by all plant ID apps in this study and scored higher in both scoring systems. Given the additional information that flowers provide over leaves (greater morphological variation, size, colour), this is not particularly surprising. Mann-Whitney U pairwise tests indicated that flowers scored significantly higher than leaves in scoring system A (p = 2.62 e^-08^). Flowers also achieved higher mean scores, higher minimum scores and lower variance was recorded across all apps for leaves than flowers (Table 5).

**Table 5:** Comparison of the mean scores, variance and minimum scores between leaves and flowers across all apps used in the current study.

			Google Lens	Plant Net	Plant Snap	Leaf Snap	Seek	iNaturalist
Raw Data	Leaf	Variance	7.20	1.73	9.02	1.68	12.92	3.96
		Mean	6.40	7.54	4.49	7.50	3.91	5.99
		Minimum	0	0	0	0	0	0
	Flower	Variance	**2.36**	**0.62**	**3.29**	**0.54**	**7.11**	**1.54**
		Mean	**7.27**	**7.75**	**6.41**	**7.73**	**6.36**	**6.45**
		Minimum	0	**4**	0	**4**	0	0
Averaged Data	Leaf	Variance	4.52	0.64	5.15	0.76	5.70	1.41
		Mean	6.12	7.50	4.15	7.46	3.65	5.92
		Minimum	0	4.8	0	4.2	0	2.6
	Flower	Variance	**1.08**	**0.32**	**1.20**	**0.29**	**2.55**	**0.42**
		Mean	**7.26**	**7.80**	**6.45**	**7.77**	**6.51**	**6.54**
		Minimum	**3**	**4.9**	**2.5**	**5.1**	**1.6**	**4.3**

Highest values for the mean and minimum scores in the raw and averaged datasets are highlighted in **bold**. The lowest variance scores for the raw and averaged datasets are also shown in **bold**.

For scoring system B, flowers also scored higher than leaves (Table 6). There was a higher percentage of correct identifications for flowers (55.18% correct) than leaves (43.21% correct) under scoring system B. A Chi-Square analysis showed that, overall, flowers were significantly better identified (p < 0.001) than leaves.

**Table 6:** Percentage of correctly identified leaf and flower images for all species across all apps.

% Of Images analysed correctly	Google Lens	Plant Net	Plant Snap	Leaf Snap	Seek	iNaturalist
**Leaves**	55.00	80.36	17.14	77.14	22.86	6.79
**Flowers**	**67.86**	**88.21**	**35.71**	**83.57**	**52.14**	**3.57**

## Discussion

There was a significant difference between each plant ID app across the entire data set, with Plant Net being the most successful app at accurately identifying native Irish herbaceous angiosperms in the field. This was closely followed by Leaf Snap. Google Lens and iNaturalist both did well and Plant Snap and Seek were the lowest preforming apps in this study.

This ranking of the apps was consistent across all families. The higher-ranking apps scored the highest points in all families and the lower ranking apps scored the lowest points. Plant Net is the leading app across all families, scoring significantly higher than the lowest scoring app, Plant Snap (*p* < 0.0001). The consistency of the apps across different families suggests that the higher scoring plant ID apps (Plant Net and Leaf Snap) can reliably identify plants with a variety of morphological features and characteristics because the three largest families in the study (Asteraceae, Fabaceae and Rosaceae) are morphologically distinct from each other. However, even with these high-scoring apps, they still failed to correctly identify plants between 12 and 23% of the time, highlighting that even the well-preforming apps continue to need development to improve accuracy.

Plant Net correctly identified 88.21% of flower images and 80.36% of leaves to a species level on the first attempt in this study. This contrasts with the findings of Jones [15] and Bilyk et al. [21]. Neither study ranked Plant Net highest out of their selected apps, which included Google Lens, Seek and Plant Snap. Jones [15] analysed 38 images that included plant organs and whole plants, using nine free-to-use plant ID apps. These images captured a variety of life forms and plant organs including flowers, leaves, bark, herbs, monocots and woody plants. They also utilised multiple scoring systems, taking taxonomic accuracy and single scores into account for their analysis. Scores were awarded for correctly identifying plants to a species, genus or family level while also awarding scores to “similar” species, genera or families. In their analysis, Google Lens and Seek scored higher than Plant Net and the other shared app, Plant Snap. Plant Snap did not perform better than Plant Net in Jones [15], but was the next worse app, scoring higher than several others. This highlights how subjective scoring systems may alter results and apps may rank differently between studies.

Plant ID apps have considerable potential in a wide range of areas. For example, Bilyk *et al*. [21] evaluated apps for their proposed use in schools to assist in education. Apps were ranked based on ease of installation and use, something that was not considered as part of the scoring system in the current study nor by Jones [15], as well as their ability to identify plants. Plant Net ranked highly for ease of installation and user-friendliness but did not identify plants to an acceptable standard of accuracy according to Bilyk et al. [21] having correctly identified 55% of plants correctly. Google Lens far outperformed all other apps, which included Plant Net, Leaf Snap, Plant Snap, iNaturalist and Seek, correctly identifying 92.6% of plants. Bilyk *et al*. [21] stated clearly that they would not recommend the use of Plant Net due to a lack of identification accuracy, which is particularly interesting as this app was the highest scoring app in the current study. Conversely, Plant Net has been shown to have potential in research applications. Yang et al. [22] found that it could identify different phenotypes of Arabidopsis with over 96% accuracy depending on the phenotype. Similarly Li et al. [23] showed that Plant Net was highly accurate at aiding in identifying varieties of tomato, tobacco and sorghum from plant organs and likely to be useful in further developing accurate phenotyping methodologies.

Universal, objective ranking of plant ID apps is difficult due to the variation of app functions and requirements of the user. For example, different plant ID apps may be preferable for individuals of varying ages, and this must be reflected upon when deciding which app is the best one to use. Looking at the results of the current study, one would be inclined to recommend Plant Net or Leaf Snap as the best plant ID apps. However, even though Plant Net and Leaf Snap reported the highest accuracy, these may be inappropriate for some users. Due to the vast range of information and botanical terminology provided by these apps, they may be deemed less accessible than other apps, particularly for people who are not familiar with botanical terminology. Seek’s encouragement of users via challenges and achievements, coupled with a simplistic and colourful design could makes it appealing to younger users [23], but its lower accuracy in this study raises concerns. Google Lens’ widespread availability and generally “good” scores also make it potentially quite useful for the casual user. As highlighted above, the right app varies depending on the aim of use—with Plant Net being useful for phenotyping [22, 23] but having preformed less well when assessed for teaching purposes [21].

**Fig 2:**
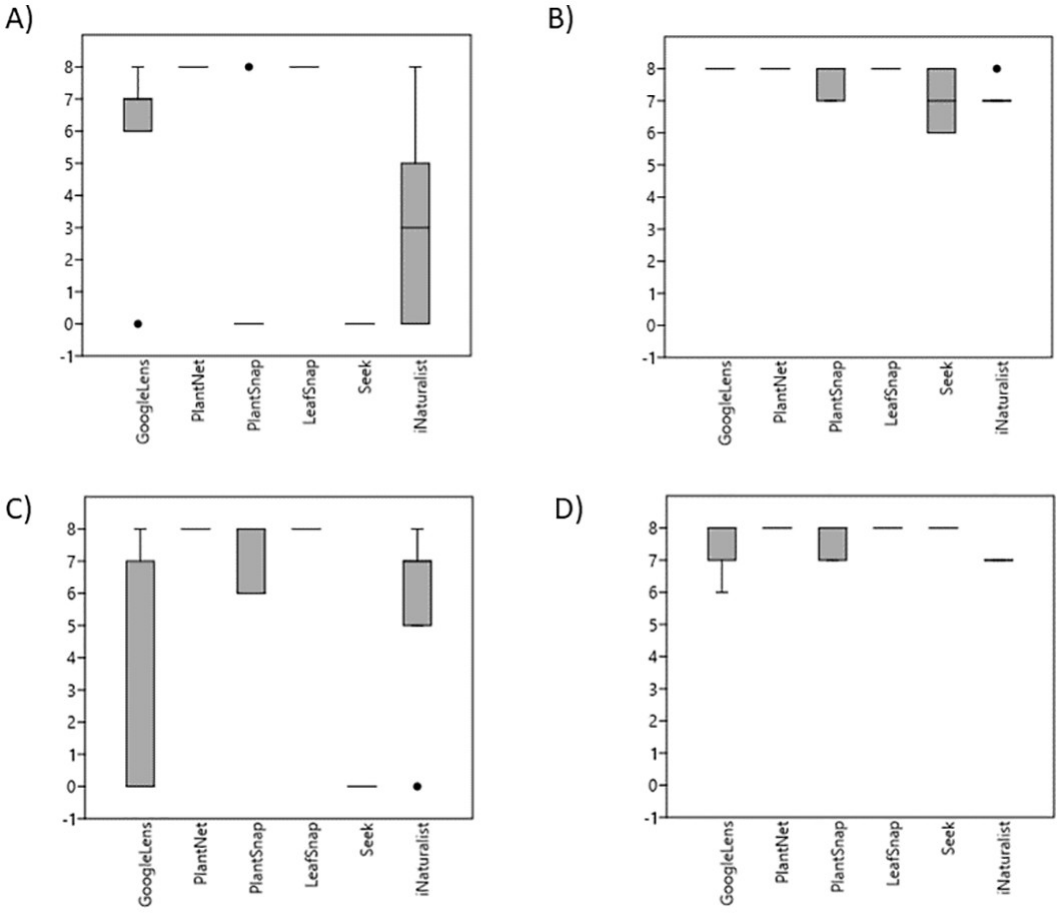
Leaf and flower scores for the toxic plants used in the current study (A) Foxglove (*Digitalis purpurea*) leaf raw data (B) Foxglove (*Digitalis purpurea*) flower raw data (C) Enchanter’s nightshade (*Circaea lutetiana*) leaf raw data (D) Enchanter’s nightshade (*Circaea lutetiana*) flower raw data. The line bisecting each box represents the median sore achieved by that app.

Apps offer opportunities not just for engaging people with plants and education but potentially for determining if a plant may be problematic. For example, Otter *et al*. [16] tested how well three apps including Plant Net and Plant Snap, were able to identify 17 toxic plants. Plant Snap was the least accurate of the three identifying only one of 17 poisonous plants (5.9%), with Plant Net scoring higher, identifying eight of 17 (47%) of the test plants. Toxic plants were not the focus of the current study but two were included: *Digitalis purpurea* and *Circaea lutetiana*. Plant Net scored higher than Plant Snap for identifying flowers and leaves for both plants (Fig 2) but no app consistently accurately identified either—highlighting that they should not be trusted to identify toxic species.

The study highlights that there is considerable variation within and between identification apps for identifying plants. It is also difficult to compare across studies due to a range of different scoring systems being used. The current study provides two reproducible scoring systems that can be used across apps and across taxonomic groups to investigate the ability of identification apps to accurately identify not only plants, but organisms in general.

## Conclusion

Overall, none of the apps in this study achieved a highly consistent level of accuracy (e.g. over 90%) but several were quite good at identifying herbaceous plants. The technology is also improving all the time, and it is therefore likely that apps will only continue to become more accurate. There is significant potential for the use of some generalist ID apps to help people identify plants. They have potential to be of use in a wide range of situations from curious gardeners, to trainee environmental professionals to teaching. However, apps should be considered an aid to plant identification and not assumed to be correct, particularly if the plant in question might be toxic or otherwise harmful.

## Supporting information

S1 Data (XLSX)
